# Molecular Evolution of Threonine Dehydratase in Bacteria

**DOI:** 10.1371/journal.pone.0080750

**Published:** 2013-12-04

**Authors:** Xuefei Yu, Ye Li, Xiaoyuan Wang

**Affiliations:** 1 State Key Laboratory of Food Science and Technology, Jiangnan University, Wuxi, China; 2 Key Laboratory of Industrial Biotechnology of Ministry of Education, Jiangnan University, Wuxi, China; 3 Synergetic Innovation Center of Food Safety and Nutrition, Jiangnan University, Wuxi, China; Beijing Institute of Microbiology and Epidemiology, China

## Abstract

Threonine dehydratase converts L-threonine to 2-ketobutyrate. Several threonine dehydratases exist in bacteria, but their origins and evolutionary pathway are unknown. Here we analyzed all the available threonine dehydratases in bacteria and proposed an evolutionary pathway leading to the genes encoding three different threonine dehydratases CTD, BTD1 and BTD2. The ancestral threonine dehydratase might contain only a catalytic domain, but one or two ACT-like subdomains were fused during the evolution, resulting BTD1 and BTD2, respectively. Horizontal gene transfer, gene fusion, gene duplication, and gene deletion may occur during the evolution of this enzyme. The results are important for understanding the functions of various threonine dehydratases found in bacteria.

## Introduction

**Figure 1:**
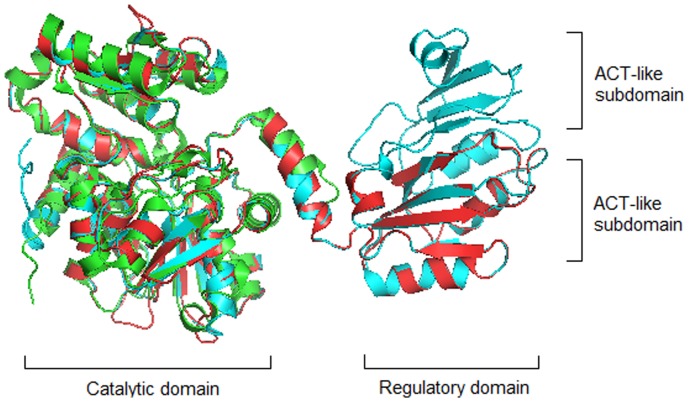
Structure comparison of BTD2 (1TDJ) in *E. coli*, BTD1 in *B. subtilis* and CTD (2GN2) in *S. typhimurium*. Two domains in BTD1 and BTD2 are separated by a middle linker. The larger domain on the left is the catalytic domain, and the smaller one on the right is the regulatory domain composed of ACT-like subdomains. CTD (shown in green) contains only the catalytic domain; BTD1 (shown in red) contains the catalytic domain and one ACT-like subdomain; BTD2 (shown in blue) contains the catalytic domain and two ACT-like subdomains.

There are usually two types of threonine dehydratase (TD) in bacteria: the biosynthetic threonine dehydratase (BTD) and the catabolic threonine dehydratase (CTD). They both could convert L-threonine to 2-ketobutyrate, BTD functions in the biosynthetic pathway of L-isoleucine when bacteria grow under the aerobic condition, while CTD plays a role in the degradation of L-threonine to propionate when bacteria grow under the anaerobic condition [1]. BTD usually contains an N-terminal catalytic domain and a C-terminal regulatory domain, while CTD usually contains only the catalytic domain. Sequence and structure analyses have revealed that the C-terminal regulatory domain of BTD is composed of one or two ACT-like subdomains (Fig. 1). BTD containing two ACT-like subdomains (BTD2) encoded by the gene *ilvA* in *Escherichia coli* is the key enzyme for L-isoleucine biosynthesis, and its activity is inhibited by the end product L-isoleucine but could be countered by L-valine, the product of a competing biosynthetic pathway [2]. BTD containing one ACT-like subdomain (BTD1) encoded by *ilvA* in *Bacillus subtilis* could be inhibited by L-isoleucine or by high concentrations of L-valine [3]. CTD encoded by the gene *tdcB* in *Salmonella typhimurium* is insensitive to L-isoleucine or L-valine, but its activity could be activated by AMP and CMP [4]. These examples indicate that the function of TD is closely related to the number of ACT-like subdomains it contains.

The sequence and/or structure of several TDs in bacteria have been characterized [2], [5], [6], but the differences on the sequence and structure of CTD, BTD1 and BTD2 are not fully understood. In this study, we analyzed the amino acid sequences of all the available TDs in bacteria, and proposed an evolutionary pathway leading to the genes encoding CTD, BTD1 and BTD2 in the present bacteria.

## Materials and Methods

### Sequential and structural alignment of CTD and BTD

There are 15120 TD sequences in the protein database of NCBI. The number of amino acids in these TDs is mainly around 350, 400 or 510. Because CTD usually contains less amino acids than BTD, we assume that the TDs containing about 350 amino acids are CTD. Thus, all TDs were divided into two groups: BTDs which contain more than 360 amino acids, and CTDs which contain less than 360 amino acids. One BTD and/or CTD sequence was chosen from each genus, and as a result, 546 BTDs and 328 CTDs were chosen. These TDs were further confirmed by using Conserved Domain Architecture Retrieval Tool (CDART) in NCBI [7] to check if they contain the ACT-like subdomain. The sequence alignments of these BTDs and CTDs were performed by using ClustalX 2.1 [8], and the logos were generated by using Weblogo 3 web service [9] (http://weblogo.threeplusone.com/create.cgi).

The crystal structure of BTD2 (1TDJ) from *E. coli* and CTD (2GN2) from *S. typhimurium* were obtained from PDB database [10]. The structure of BTD1 coded by gene *ilvA* from *B. subtilis* was modeled by using SWISS-MODEL Web server [11] with default parameters. These structures were used to build the comparison model by PyMol. The crystal structures of *E. coli* BTD2 and *S. typhimurium* CTD were further pairwise aligned by using FATCAT web service [12] with flexible model, and the structural alignment of the PLP binding sites and the substrate binding sites were performed by using PyMol.

### Distribution of species containing TD and construction of phylogenetic trees

The distribution of species containing TDs in nature were obtained from the UniProtKB database (http://www.uniprot.org/browse/uniprot/by/taxonomy/?query=ec%3A4.3.1.19) [13]. In this database 3607 species were found to contain TDs, they include 3504 species in Bacteria and 103 species in Archaea and Eukaryotes. Because the 3504 bacterial species are mainly distributed in Proteobacteria (1803 species), Firmicutes (1285 species) and Actinobacteria (280 species), representative species were selected from these three phyla for further study. Sequence analysis showed that TDs from the stains within the same species are highly conserved, thus we selected one TD sequence from each species to construct the phylogeny. 1–5 representative species were selected in the same order within α-, β-, δ-, ε- and γ-proteobacteria, and in the same class in Firmicutes and Actinobacteria. Total 82 species were selected. TDs in these 82 representative species were searched by using BLASTp with default parameters, and the sequence of *E. coli* BTD2 encoded by *ilvA* was used as the query. The representative species and the TDs they contain are listed in Table S1. These TDs were divided into groups of BTD1, BTD2 and CTD, based on the number of ACT-like subdomains they contain which were determined by CDART analysis. 16s rDNA sequences of these 82 strains were collected from Ribosomal Database Project (RDP) database [14]. The alignment of multiple sequences was performed by using ClustalX 2.1. Phylogenetic trees of protein sequences and 16s rDNA sequences were performed by using Mega 5 [15] software and the neighbor-joining methods.

## Results

### Catalytic domains of all CTDs and BTDs are conserved

**Figure 2:**
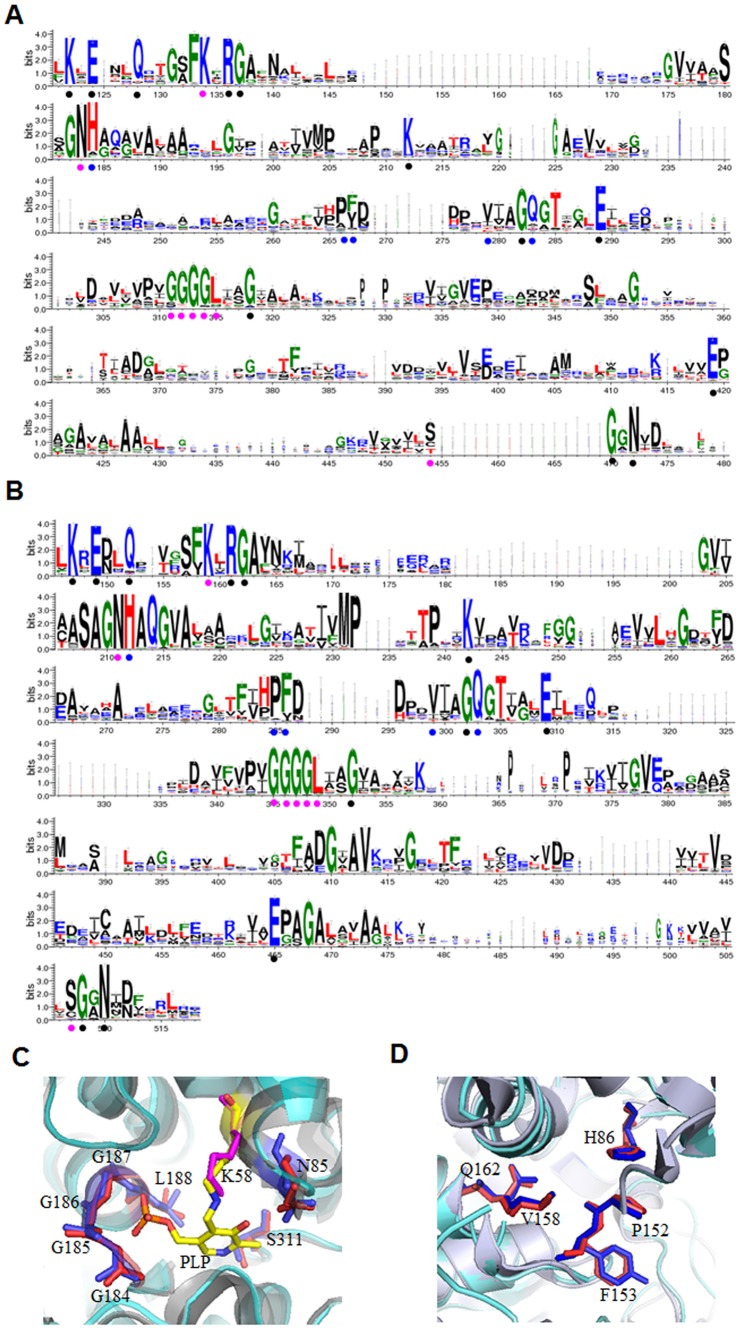
Sequence alignment of CTDs and BTDs and structure alignment of BTD2 (1TDJ) and CTD (2GN2). A. The sequence alignment of CTDs from 328 species of bacteria. B. The sequence alignment of BTDs from 546 species of bacteria. The PLP binding sites and the substrate binding sites are labelled by purple and blue dots, respectively. The other highly conserved residues are labelled by black dots. C. The aligned structure of PLP binding sites of BTD2 and CTD. D. The aligned structure of substrate binding sites of BTD2 and CTD. The amino acid residues directly involved in PLP binding sites and the substrate binding sites are shown in sticks. Residues from CTD are shown in blue and residues from BTD are shown in red. The residues are labled accoding to the sequence of CTD coded by *tdcB* in *S. typhimurium* [4].

Both BTD and CTD could convert L-threonine to 2-ketobutyrate. To understand their difference and evolutionary relationship the sequence and structure of BTDs and CTDs were analyzed. The sequence logos of CTD (Fig. 2A) and BTD (Fig. 2B) were generated from 328 bacterial CTDs and 546 bacterial BTDs. Because TD belongs to pyridoxal-5′-phosphate (PLP)-dependent enzyme type II family [16], [17], the conserved amino acids for binding PLP were found in both logos of CTDs (K134, N183, G311, G312, G313, G314, L315, S454) and BTDs (K159, N211, G345, G346, G347, G348, L349, S507). The conserved amino acids for substrate binding sites were also found in both logos of CTDs (H184, P266, F/Y267, V279, Q283) and BTDs (H212, P285, F/Y286, V299, Q303) [4]. Other highly conserved residues found in both logos include K122, E124, Q128, R136, G137, K212, G282, E289, G318, E419, G470, N472 for CTD (Fig. 2 A) and K147, E149, Q152, R161 G162, K242, G302, E309, G352, E465, G508, N510 for BTD (Fig. 2B), corresponding to the residues K47, E49, Q52, R60, G61, K113, G161, E168, G191, E282, G312, N314 in CTD encoded by *tdcB* in *S. typhimurium*. The correlation between the phylogenetic relationship and conservation of certain key residues in TDs, and the function of some highly conserved residues need to be further studied.

Structure of a specific BTD (1TDJ) encoded by *ilvA* in *E. coli* and a specific CTD (2GN2) encoded by *tdcB* in *S. typhimurium* were aligned; the RMSD (root mean square deviation) was 1.90 Å with 321 N-terminal residues aligned. As shown in Fig. 2C and D, the key amino acids at both the PLP binding sites (K58, N85, G184, G185, G186, G187, L188, S311) and the substrate binding sites (H86, P152, F153, V158, Q162) are all superimposed coincidently. The highly conserved structure and sequence of BTD and CTD suggest that the N-terminal of CTD and BTD should be evolved from the same ancestor [18].

### Phylogenetic analysis suggests that gene fusion, duplication and deletion events have occurred during TD evolution

Based on UniProtKB database, TDs are widely distributed in 3,607 species: 97% in Bacteria, 1.6% in Eukaryotes and 1.4% in Archaea. Bacterial TDs are mainly distributed in Proteobacteria (51%), Firmicutes (37%), and Actinobacteria (8%). Therefore, 82 strains were selected from these three phyla of bacteria as representative species for the phylogenetic analysis: 48 strains from Proteobacteria, 17 strains from Firmicutes, and 17 strains from Actinobacteria (Table S1).

**Figure 3:**
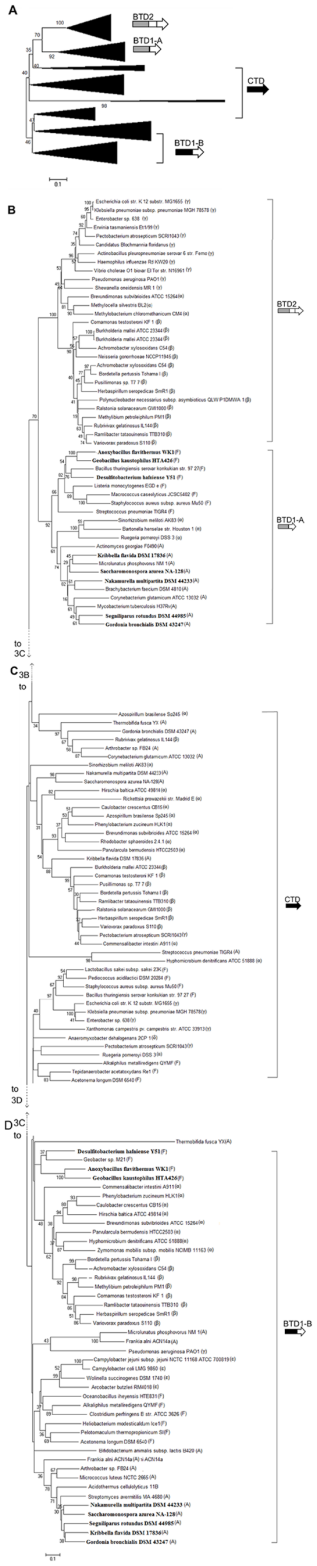
Phylogenetic tree based on the amino acid sequences of TDs from 82 representative species. Genes encoding the enzymes are represented by arrows. The overall structure of the phylogenetic tree is shown in A. Because it is too big to show in a single page, the detail structure of the phylogenetic tree is divided into three panels (B, C and D). The connecting point of the tree segments in the three panels is marked with a broken line. The strains shown in bold contain both genes encoding for BTD1-A and BTD1-B. α, β, δ, ε, γ, F and A indicate α-proteobacteria, β-proteobacteria, δ-proteobacteria, ε-proteobacteria, γ-proteobacteria, Firmicutes and Actinobacteria, respectively. The tree was constructed with the MEGA 5 software using the neighbor-joining method and 1000 bootstrap replicates.

A phylogenetic tree was constructed using the protein sequences of TD from the 82 bacterial species (Fig. 3). Overall there were major four clusters in the tree: one CTD cluster, two BTD1 clusters (BTD1-A and BTD1-B) and one BTD2 cluster (Fig. 3A). In this study, TD sequences for constructing the phylogenetic tree were selected from a wide range of species and the length of BTDs and CTDs are quite different. Therefore, some bootstrap values on the tree are lower than 50. BTD2 was found mainly in species of β- and γ- Proteobacteria, and a few species of α-Proteobacteria (Fig. 3B); BTD1-A was found mainly in species of Firmicutes, Actinobacteria and a few species of α-Proteobacteria (Fig. 3B); BTD1-B and CTD were found in species of all the three phyla: Proteobacteria, Firmicutes and Actinobacteria (Fig. 3C and D). The finding of two distinct BTD1 clusters, BTD1-A and BTD1-B, is interesting. There were 8 species of Firmicutes and Actinobacteria (shown in bold in Fig. 3) containing both BTD1-A and BTD1-B, suggesting that gene duplication of BTD1 might occur in the bacteria. According to the tree, BTD1-A cluster is much closer to BTD2 cluster than to BTD1-B, while BTD1-B cluster is much closer to CTD cluster. Based on these data, CTD might be the common ancestor for all the TDs, and BTD1 and BTD2 might be the gene fusion product of ancestral CTD and ACT-like subdomains because the combination of different domains is an important mechanism for the evolution of multidomain proteins [19]; BTD2 might be derived from ancestral BTD1-A during evolution because it is much closer to BTD1-A cluster than to BTD1-B cluster in the phylogenetic tree. Phylogeny trees were constructed using sequences of ACT-like subdomain of BTD1 and each of the two ACT-like subdomains of BTD2, and the results showed that the first ACT-like subdomain of BTD2 is closer to the ACT-like subdomain of BTD1 than the second ACT-like subdomain of BTD2. This does not mean that the second ACT-like subdomain of BTD2 was generated from a new ACT subdomain, because it could also be duplicated from the ACT-like subdomain of BTD1, considering the duplicated sequences of a protein are usually highly divergent to avoid the misfolding. Moreover, though the regulatory domains of TDs have close structural and functional relationships with ACT family domains [20]–[21], they have little sequence similarity with ACT family domains, and could not be assigned by PSI-BLAST as ACT family. Thus, the regulatory domains of TDs are named as ACT-like subdomains. Therefore, the second ACT-like subdomain of BTD2 is more likely the result of a duplication of the ACT-like subdomain of BTD1 rather than a fusion of a new ACT subdomain. Since BTDs also exist in Eukaryotes and Archaea, the fusion of CTD and ACT-like domain could be happened before the divergence of three kingdoms.

**Figure 4:**
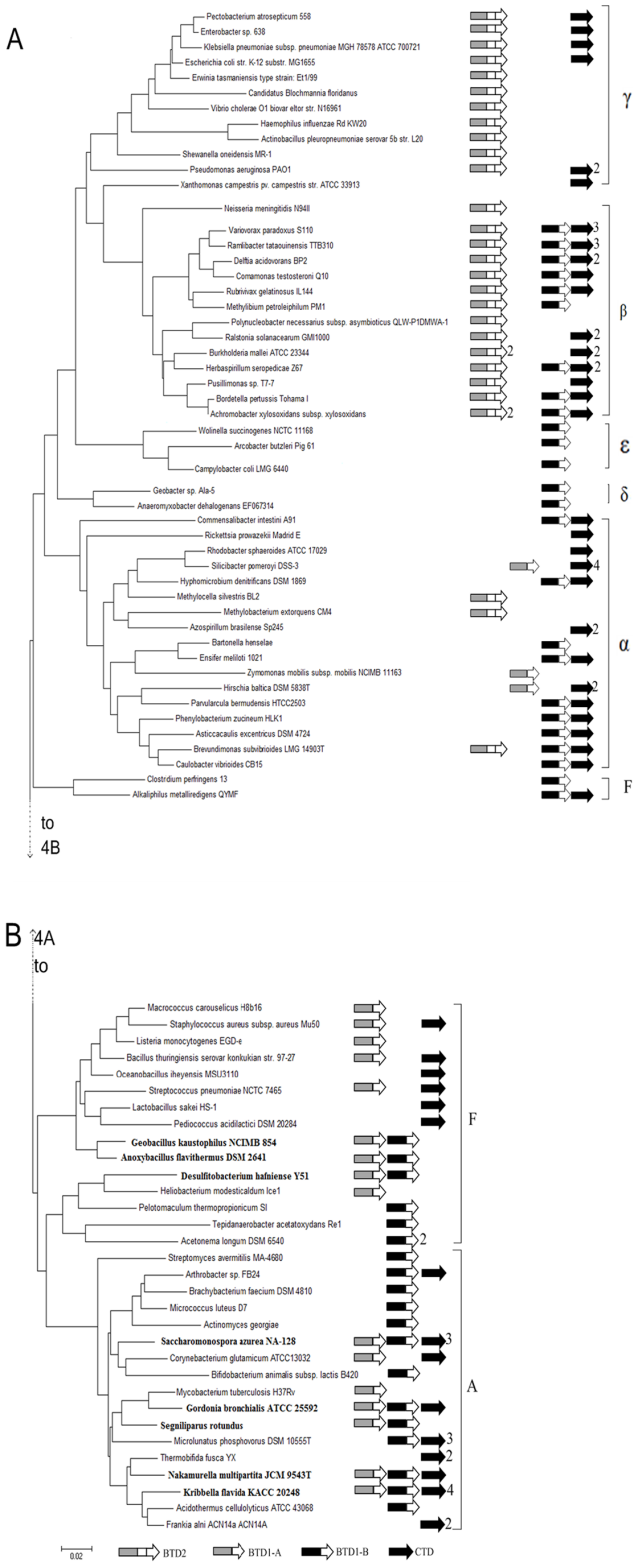
Phylogenetic tree based on 16S rDNA sequences showing the phylogenetic distribution of the TD enzyme. The phylogenetic tree was constructed with MEGA 5 software using sequences from RDP database. Because it is too big to show in a single page, the structure of the phylogenetic tree is divided into two panels (A and B). The connecting point of the tree segments in the two panels is marked with a broken line. The scale bar indicates 0.02 change per nucleotide. The arrows at the right represent the TDs that could exist in the bacterium and the numbers next to the arrow show the number of genes that might encode the TD.

Fig. 4 shows the phylogenetic tree constructed from the sequences of 16s rDNA of the 82 bacterial strains (Table S1). The arrows next to the species indicate CTD, BTD1-A, BTD1-B or BTD2. BTD1 encoding genes were found in all three phyla except for γ-Proteobacteria. Both BTD1-A and BTD1-B were found in 8 bacterial species (shown in bold), but only one of them was found in other species, suggesting the deletion event of BTD1-A or BTD-1B might happen after the duplication event of BTD1. BTD2 was found in almost every species of β- and γ-proteobacteria, but only in 3 species of α-proteobacteria. This suggests that BTD2 might generate within the ancestor of β- and γ-proteobacteria after its divergence from α-proteobacteria, and BTD2 existing in the 3 species of α-proteobacteria could be generated by horizontal gene transfer from species of β- or γ-proteobacteria (Fig. 4A). Although most of the 82 strains exist more than two TDs, BTD2 and BTD1-A were never found in the same strain, suggesting that BTD2 should be derived from the ancestral BTD1-A by fusing with another duplicated ACT-like subdomain. BTD1-B and BTD2 were found in some species of β-proteobacteria, but only BTD2 encoding genes were found in γ-proteobacteria, suggesting that BTD1-B might be deleted in some species after BTD2 was evolved. CTD, BTD1-B and BTD2 were all found in 8 bacterial strains of Proteobacteria but only one or two of them found in other strains, strongly suggesting that the deletion events might happen for TDs in bacteria during the evolution.

## Discussion

**Figure 5:**
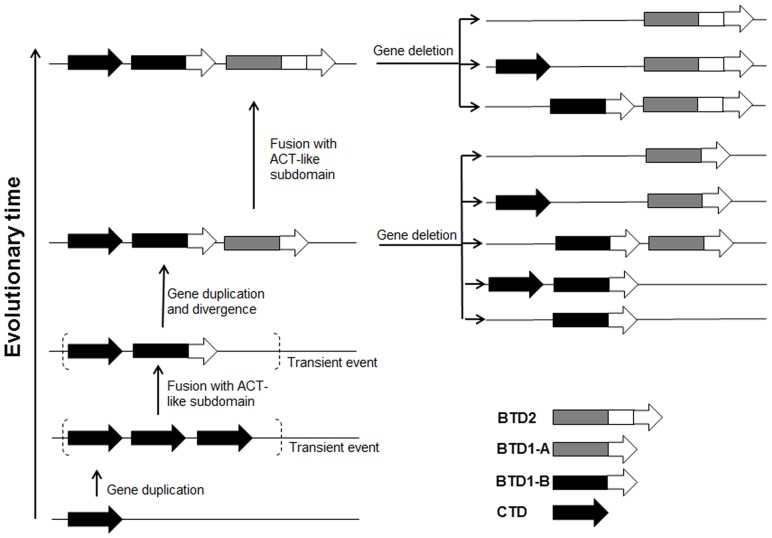
Evolutionary model proposed for the evolution of genes encoding TDs in bacteria. Genes encoding the enzymes are represented by arrows.

Based on the homology and phylogenetic analysis, an evolutionary model for TDs was proposed (Fig. 5). The ancestor possessed only a single copy of gene encoding CTD containing only the catalytic domain. Later the gene was duplicated, and the redundant copy was fused with a DNA fragment encoding for ACT-like subdomain, producing the gene encoding for BTD1-B. Then this gene was duplicated, generating a copy encoding for BTD1-A. With the divergence of new species, one or two of the genes encoding for CTD, BTD1-A and BTD1-B were deleted from the genome. The similar duplication and deletion events were also found for the *lpxH* gene in Kdo_2_ lipid A biosynthesis pathway [22]. The gene *lpxH* was duplicated within Proteobacteria, and one of them was lost along with new species generation. Within the ancestor of some species of Proteobacteria, the ACT-like subdomain of BTD1-A might be duplicated, generating BTD2. With the divergence of new species, the gene encoding for CTD, or BTD1-B were deleted from the genome. Two copies of BTD2 were observed in one species of Proteobacteria, suggesting that the duplication of BTD2 could also occur.

Our proposed evolutionary model of TD is consistent with the published theories, which suggest that organisms prefer to generate new genes encoding multiple domain proteins from the pre-existing genes [19], [23], [24], and new enzymes are usually evolved from enzymes with similar biochemical function rather than in the same biosynthetic pathway [25]–[28]. CTD exists not only in bacteria, but also in plants and yeast [29]–[33], suggesting that the pathway of L-threonine degradation may exist in the ancestral cell before the divergence of the three kingdoms. In the primordial soup where organic compounds were rich, the ancestral cell might have more catabolic pathways than biosynthetic pathways, therefore, it might only need CTD for gaining energy under the anaerobic condition [23]. With the increase of the number of primordial cells, the prebiotic supply of amino acids might be exhausted, and 2-ketobutyrate produced by CTD might also be used for L-isoleucine biosynthesis. For better adapting the environment, BTD were created in modern bacterial species by combining CTD and ACT-like subdomain to satisfy the necessary regulation of L-isoleucine and/or L-valine [34]. ACT family domain is wildly conserved in bacteria and evolutionarily mobile. It is always combined with other domains to provide easily regulated enzymes [21], [35].

The interaction between different domains may lead the enzyme easier to fold correctly [36]. Thus BTD1 or BTD2 which contains both the catalytic domain and the ACT-like subdomain might be more stable than CTD which contains only the catalytic domain. The activity of BTD2 might be regulated more easily than that of BTD1 because BTD2 contains one more ACT-like subdomain than BTD1 [3]. Flexibility is one important reason for protein evolution, and the mechanical flexibility of proteins are critical for their functions [37]. More flexible the structure of an enzyme is more easily its activity could be regulated [38], [39]. This suggests that the structure of BTD2 may be more flexible than BTD1, and BTD2 might be evolved to benefit bacteria to adapt the more complex environment [38], [40]. As the activity of BTD is inhibited by the end product L-isoleucine, constructing feedback resistant BTD has been used to increase the L-isoleucine production in industrial fermentation [41]–[43]. CTD encoded by *tdcB* from *E. coli* has been overexpressed in *C. glutamicum* to improve the production of L-isoleucine [44], [45]. Our results suggest that directly removing the regulatory domain of an enzyme might be an effect way to obtain a feedback-resistant enzyme for the metabolic engineering in bacteria.

## Supporting Information

Table S1 The representative bacterial species used in the phylogenetic analysis of TDs.
(DOCX)
