# Supplementary material for: Molecular Evolution of Threonine Dehydratase in Bacteria
Source: PLoS One. 2013 Dec 4;8(12):e80750. doi: 10.1371/journal.pone.0080750 (PMC3851459; doi:10.1371/journal.pone.0080750)
Supplement: Table S1 — The representative bacterial species used in the phylogenetic analysis of TDs. (DOCX) [file pone.0080750.s001.docx]

Molecular evolution of threonine dehydratase in bacteria

Xuefei Yu^1,2^, Ye Li^2^, Xiaoyuan Wang^1,2^

^1^State Key Laboratory of Food Science and Technology, Jiangnan University, Wuxi 214122, China; ^2^Key Laboratory of Industrial Biotechnology of Ministry of Education, School of Biotechnology, Jiangnan University, Wuxi 214122, China.

Running title: Molecular evolution of threonine dehydratase

**Corresponding author：**

Prof. Xiaoyuan Wang

State Key Laboratory of Food Science and Technology

Jiangnan University

1800 Lihu Avenue

Wuxi 214122

China

Tel: +86 510 85329239

Fax: +86 510 85329239

E mail: [xiaoyuanwang@hotmail.com](mailto:xiaoyuanwang@hotmail.com)

**Supplementary Tables**

**Table S1: The representative bacterial species used in the phylogenetic analysis of TDs.**

| ***Species*** | **GI** | **AA numbers** | **TD type** |
| --- | --- | --- | --- |
| **γ-proteobacteria** |  |  |  |
| *Pectobacterium atrosepticum* SCRI1043 | gi\|50120968 | 324 | CTD |
| *Pectobacterium atrosepticum* SCRI1043 | gi\|50120505 | 334 | CTD |
| *Pectobacterium atrosepticum* SCRI1043 | gi\|50123145 | 516 | BTD2 |
| *Enterobacter sp.* 638 | gi\|146313643 | 515 | BTD2 |
| *Enterobacter sp.* 638 | gi\|146313199 | 329 | CTD |
| *Klebsiella pneumoniae subsp. pneumoniae* MGH 78578 | gi\|152972764 | 514 | BTD2 |
| *Klebsiella pneumoniae subsp. pneumoniae* MGH 78578 | gi\|152970842 | 329 | CTD |
| *Escherichia coli str. K 12 substr.* MG1655 | gi\|16131630 | 514 | BTD2 |
| *Escherichia coli str. K 12 substr.* MG1655 | gi\|16131010 | 329 | CTD |
| *Erwinia tasmaniensis* Et1/99 | gi\|188532345 | 514 | BTD2 |
| *Candidatus Blochmannia floridanus* | gi\|33520028 | 514 | BTD2 |
| *Vibrio cholerae O1 biovar El Tor str.* N16961 | gi\|15640059 | 510 | BTD2 |
| *Haemophilus influenzae* Rd KW20 | gi\|16272680 | 513 | BTD2 |
| *Actinobacillus pleuropneumoniae* serovar 6 str. Femo | gi\|303250955 | 508 | BTD2 |
| *Shewanella oneidensis* MR 1 | gi\|24375825 | 545 | BTD2 |
| *Pseudomonas aeruginosa* PAO1 | gi\|15596523 | 515 | BTD2 |
| *Pseudomonas aeruginosa* PAO1 | gi\|15596048 | 320 | CTD |
| *Xanthomonas campestris pv. campestris str.* ATCC 33913 | gi\|21232755 | 372 | CTD |
| **β-proteobacteria** |  |  |  |
| *Neisseria gonorrhoeae* NCCP11945 | gi\|194098191 | 508 | BTD2 |
| *Variovorax paradoxus* S110 | gi\|239814090 | 529 | BTD2 |
| *Variovorax paradoxus* S110 | gi\|239813168 | 409 | BTD1-B |
| *Variovorax paradoxus* S110 | gi\|239816443 | 318 | CTD |
| *Ramlibacter tataouinensis* TTB310 | gi\|337281190 | 514 | BTD2 |
| *Ramlibacter tataouinensis* TTB310 | gi\|337277740 | 402 | BTD1-B |
| *Ramlibacter tataouinensis* TTB310 | gi\|337279764 | 321 | CTD |
| *Comamonas testosteroni* KF 1 | gi\|221069093 | 517 | BTD2 |
| *Comamonas testosteroni* KF 1 | gi\|221069695 | 408 | BTD1-B |
| *Comamonas testosteroni* KF 1 | gi\|221066384 | 325 | CTD |
| *Rubrivivax gelatinosus* IL144 | gi\|383756663 | 513 | BTD2 |
| *Rubrivivax gelatinosus* IL144 | gi\|383755928 | 400 | BTD1-B |
| *Rubrivivax gelatinosus* IL144 | gi\|383757221 | 311 | CTD |
| *Methylibium petroleiphilum* PM1 | gi\|124268309 | 526 | BTD2 |
| *Methylibium petroleiphilum* PM1 | gi\|124268954 | 412 | BTD1-B |
| *Polynucleobacter necessarius subsp. asymbioticus* QLW P1DMWA-1 | gi\|145590079 | 506 | BTD2 |
| *Ralstonia solanacearum* GMI1000 | gi\|17545168 | 507 | BTD2 |
| *Ralstonia solanacearum* GMI1000 | gi\|17549735 | 323 | CTD |
| *Burkholderia mallei* ATCC 23344 | gi\|53724987 | 507 | BTD2 |
| *Burkholderia mallei* ATCC 23344 | gi\|77358938 | 372 | BTD1-B |
| *Burkholderia mallei* ATCC 23344 | gi\|229220800 | 327 | CTD |
| *Herbaspirillum seropedicae* SmR1 | gi\|300313197 | 508 | BTD2 |
| *Herbaspirillum seropedicae* SmR1 | gi\|300309629 | 326 | CTD |
| *Herbaspirillum seropedicae* SmR1 | gi\|300309398 | 401 | BTD1-B |
| *Pusillimonas sp.* T7-7 | gi\|332286609 | 502 | BTD2 |
| *Pusillimonas sp.* T7-7 | gi\|332285259 | 322 | CTD |
| *Bordetella pertussis* Tohama I | gi\|33591392 | 502 | BTD2 |
| *Bordetella pertussis* Tohama I | gi\|33591969 | 405 | BTD1-B |
| *Bordetella pertussis* Tohama I | gi\|33594303 | 322 | CTD |
| *Achromobacter xylosoxidans* C54 | gi\|317407492 | 525 | BTD2 |
| *Achromobacter xylosoxidans* C54 | gi\|317401298 | 502 | BTD2 |
| *Achromobacter xylosoxidans* C54 | gi\|317402108 | 405 | BTD1-B |
| **ε-proteobacteria** |  |  |  |
| *Wolinella succinogenes* DSM 1740 | gi\|34556581 | 403 | BTD1-B |
| *Arcobacter butzleri* RM4018 | gi\|157738232 | 401 | BTD1-B |
| *Campylobacter jejuni subsp. Jejuni* ATCC 700819 | gi\|218562456 | 403 | BTD1-B |
| **δ-proteobacteria** |  |  |  |
| *Geobacter sp.* M21 | gi\|253702372 | 402 | BTD1-B |
| *Anaeromyxobacter dehalogenans* 2CP-1 | gi\|220918689 | 403 | BTD1-B |
| **α-proteobacteria** |  |  |  |
| *Commensalibacter intestini* A911 | gi\|354593479 | 400 | BTD1-B |
| *Commensalibacter intestini* A911 | gi\|354593999 | 322 | CTD |
| *Rickettsia prowazekii str.* Madrid E | gi\|15604314 | 333 | CTD |
| *Rhodobacter sphaeroides* 2.4.1 | gi\|77462803 | 325 | CTD |
| *Hyphomicrobium denitrificans* ATCC 51888 | gi\|300023369 | 411 | BTD1-B |
|  | gi\|300024931 | 345 | CTD |
| *Methylocella silvestris* BL2 | gi\|217977980 | 505 | BTD2 |
| *Methylobacterium chloromethanicum* CM4 | gi\|218531205 | 510 | BTD2 |
| *Azospirillum brasilense* Sp245 | gi\|392382012 | 331 | CTD |
| *Azospirillum brasilense* Sp245 | gi\|392382928 | 472 | CTD |
| *Bartonella henselae str.* Houston 1 | gi\|49475814 | 421 | BTD1-B |
| *Zymomonas mobilis subsp. mobilis* NCIMB 11163 | gi\|260752311 | 412 | BTD1-A |
| *Hirschia baltica* ATCC 49814 | gi\|254292444 | 405 | BTD1-A |
| *Hirschia baltica* ATCC 49814 | gi\|254294171 | 319 | CTD |
| *Parvularcula bermudensis* HTCC2503 | gi\|304320827 | 428 | BTD1-B |
| *Parvularcula bermudensis* HTCC2503 | gi\|304320530 | 328 | CTD |
| *Phenylobacterium zucineum* HLK1 | gi\|197106806 | 413 | BTD1-B |
| *Phenylobacterium zucineum* HLK1 | gi\|197103871 | 329 | CTD |
| *Sinorhizobium meliloti* AK83 | gi\|334319286 | 323 | CTD |
| *Sinorhizobium meliloti* AK83 | gi\|334316264 | 415 | BTD1-B |
| *Brevundimonas subvibrioides* ATCC 15264 | gi\|302382076 | 506 | BTD2 |
| *Brevundimonas subvibrioides* ATCC 15264 | gi\|302381451 | 334 | CTD |
| *Brevundimonas subvibrioides* ATCC 15264 | gi\|302382059 | 400 | BTD1-B |
| *Caulobacter crescentus* CB15 | gi\|16127335 | 325 | CTD |
| *Caulobacter crescentus* CB15 | gi\|16127865 | 400 | BTD1-B |
| *Ruegeria pomeroyi* DSS 3 | gi\|56694948 | 408 | BTD1-B |
| *Ruegeria pomeroyi* DSS 3 | gi\|56696039 | 335 | CTD |
| *Asticcacaulis excentricus* CB 48 | gi\|315499308 | 400 | BTD1-B |
| *Delftia acidovorans* SPH-1 | gi\|160895565 | 415 | BTD1-B |
| *Delftia acidovorans* SPH-1 | gi\|160901116 | 517 | BTD2 |
| **Firmicutes** |  |  |  |
| *Clostridium perfringens B str*. ATCC 3626 | gi\|168212044 | 399 | BTD1-B |
| *Alkaliphilus metalliredigens* QYMF | gi\|150388377 | 317 | CTD |
| *Alkaliphilus metalliredigens* QYMF | gi\|150391142 | 414 | BTD1-B |
| *Macrococcus caseolyticus* JCSC5402 | gi\|222151106 | 420 | BTD1-A |
| *Staphylococcus aureus subsp. aureus* Mu50 | gi\|15925051 | 422 | BTD1-A |
|  | gi\|15924428 | 346 | CTD |
| *Listeria monocytogenes* EGD-e | gi\|16804030 | 422 | BTD1-A |
| *Bacillus thuringiensis serovar konkukian str.* 97-27 | gi\|49481117 | 420 | BTD1-A |
|  | gi\|49479295 | 333 | CTD |
| *Oceanobacillus iheyensis* HTE831 | gi\|23100071 | 338 | CTD |
| *Streptococcus pneumoniae* TIGR4 | gi\|15900367 | 416 | BTD1-A |
| *Streptococcus pneumoniae* TIGR4 | gi\|111657975 | 305 | CTD |
| *Lactobacillus sakei subsp. sakei* 23K | gi\|81428187 | 346 | CTD |
| *Pediococcus acidilactici* DSM 20284 | gi\|304384590 | 350 | CTD |
| *Geobacillus kaustophilus* HTA426 | gi\|56420308 | 423 | BTD1-A |
| *Geobacillus kaustophilus* HTA426 | gi\|56420126 | 402 | BTD1-B |
| *Anoxybacillus flavithermus* WK1 | gi\|212639284 | 422 | BTD1-A |
| *Anoxybacillus flavithermus* WK1 | gi\|212639226 | 402 | BTD1-B |
| *Desulfitobacterium hafniense* Y51 | gi\|89897065 | 415 | BTD1-A |
| *Desulfitobacterium hafniense* Y51 | gi\|89896990 | 406 | BTD1-B |
| *Heliobacterium modesticaldum* Ice1 | gi\|167629336 | 413 | BTD1-A |
| *Pelotomaculum thermopropionicum* SI | gi\|147676437 | 406 | BTD1-B |
| *Tepidanaerobacter acetatoxydans* Re1 | gi\|332798179 | 402 | BTD1-B |
| *Acetonema longum* DSM 6540 | gi\|338813714 | 402 | BTD1-B |
| *Acetonema longum* DSM 6540 | gi\|338812333 | 402 | BTD1-B |
| **Actinobacteria** |  |  |  |
| *Streptomyces avermitilis* MA 4680 | gi\|29829844 | 409 | BTD1-B |
| *Arthrobacter sp.* FB24 | gi\|116672038 | 412 | BTD1-B |
|  | gi\|257068109 | 310 | CTD |
| *Brachybacterium faecium* DSM 4810 | gi\|257068109 | 437 | BTD1-B |
| *Micrococcus luteus* NCTC 2665 | gi\|239917042 | 443 | BTD1-B |
| *Actinomyces georgiae* F0490 | gi\|394765736 | 429 | BTD1-B |
| *Saccharomonospora azurea* NA-128 | gi\|381163023 | 401 | BTD1-B |
| *Saccharomonospora azurea* NA-128 | gi\|381162152 | 434 | BTD1-A |
| *Saccharomonospora azurea* NA-128 | gi\|381164986 | 320 | CTD |
| *Corynebacterium glutamicum* ATCC 13032 | gi\|23308897 | 436 | BTD1-A |
|  | gi\|19552203 | 310 | CTD |
| *Bifidobacterium animalis subsp. lactis* B420 | gi\|387821454 | 416 | BTD1-B |
| *Mycobacterium tuberculosis* H37Rv | gi\|15608697 | 429 | BTD1-A |
| *Gordonia bronchialis* DSM 43247 | gi\|262201575 | 400 | BTD1-B |
| *Gordonia bronchialis* DSM 43247 | gi\|262202883 | 457 | BTD1-A |
| *Gordonia bronchialis* DSM 43247 | gi\|262201700 | 310 | CTD |
| *Segniliparus rotundus* DSM 44985 | gi\|296394061 | 403 | BTD1-B |
| *Segniliparus rotundus* DSM 44985 | gi\|296393551 | 443 | BTD1-A |
| *Microlunatus phosphovorus* NM 1 | gi\|336115890 | 448 | BTD1-B |
| *Microlunatus phosphovorus* NM 1 | gi\|336120106 | 344 | CTD |
| *Thermobifida fusca* YX | gi\|72160630 | 428 | CTD |
| *Thermobifida fusca* YX | gi\|72162142 | 310 | CTD |
| *Nakamurella multipartita* DSM 44233 | gi\|258655440 | 420 | BTD1-A |
| *Nakamurella multipartita* DSM 44233 | gi\|258654539 | 403 | BTD1-B |
| *Nakamurella multipartita* DSM 44233 | gi\|258655142 | 316 | CTD |
| *Kribbella flavida* DSM 17836 | gi\|284028621 | 322 | CTD |
| *Kribbella flavida* DSM 17836 | gi\|284028263 | 423 | BTD1-A |
| *Kribbella flavida* DSM 17836 | gi\|284033501 | 401 | BTD1-B |
| *Acidothermus cellulolyticus* 11B | gi\|117929095 | 413 | BTD1-B |
| *Frankia alni* ACN14a | gi\|111220612 | 352 | CTD |
| *Frankia alni* ACN14a | gi\|111223290 | 340 | CTD |
